# The contribution of SNAT1 to system A amino acid transporter activity in human placental trophoblast

**DOI:** 10.1016/j.bbrc.2010.06.051

**Published:** 2010-07-16

**Authors:** M. Desforges, S.L. Greenwood, J.D. Glazier, M. Westwood, C.P. Sibley

**Affiliations:** Maternal and Fetal Health Research Centre, Developmental Biomedicine, School of Medicine, Manchester Academic Health Sciences Centre, University of Manchester, St. Mary’s Hospital, Level 5-Research, Oxford Road, Manchester M13 9WL, United Kingdom

**Keywords:** MeAIB, SNAT, Syncytiotrophoblast, Cytotrophoblast, siRNA

## Abstract

System A-mediated amino acid transport across the placenta is important for the supply of neutral amino acids needed for fetal growth. All three system A subtypes (SNAT1, 2, and 4) are expressed in human placental trophoblast suggesting there is an important biological role for each. Placental system A activity increases as pregnancy progresses, coinciding with increased fetal nutrient demands. We have previously shown SNAT4-mediated system A activity is higher in first trimester than at term, suggesting that SNAT1 and/or SNAT2 are responsible for the increased system A activity later in gestation. However, the relative contribution of each subtype to transporter activity in trophoblast at term has yet to be evaluated. The purpose of this study was to identify the predominant subtype of system A in cytotrophoblast cells isolated from term placenta, maintained in culture for 66 h, by: (1) measuring mRNA expression of the three subtypes and determining the Michaelis–Menten constants for uptake of the system A-specific substrate, ^14^C-MeAIB, (2) investigating the contribution of SNAT1 to total system A activity using siRNA. *Results:* mRNA expression was highest for the SNAT1 subtype of system A. Kinetic analysis of ^14^C-MeAIB uptake revealed two distinct transport systems; system 1: *K*_m_ = 0.38 ± 0.12 mM, *V*_max_ = 27.8 ± 9.0 pmol/mg protein/20 min, which resembles that reported for SNAT1 and SNAT2 in other cell types, and system 2: *K*_m_ = 45.4 ± 25.0 mM, *V*_max_ = 1190 ± 291 pmol/mg protein/20 min, which potentially represents SNAT4. Successful knockdown of SNAT1 mRNA using target-specific siRNA significantly reduced system A activity (median 75% knockdown, *n* = 7). *Conclusion:* These data enhance our limited understanding of the relative importance of the system A subtypes for amino acid transport in human placental trophoblast by demonstrating that SNAT1 is a key contributor to system A activity at term.

## Research highlights

► mRNA levels for SNAT1 are higher than other system A subtype mRNAs in primary human cytotrophoblast. ► SNAT1 knockdown in cytotrophoblast cells significantly reduces system A activity. ► SNAT1 is a key contributor to system A-mediated amino acid transport in human placenta.

## 1 Introduction

It is well established that system A-mediated transport of amino acids across the placenta is important for maintaining normal fetal growth [1]. In humans, placental system A activity increases as pregnancy progresses [2], coinciding with increased fetal nutrient demands, and is downregulated in pregnancies complicated by fetal growth restriction (FGR) [3,4]. System A is a Na^+^-dependent amino acid transporter that actively transports small, zwitterionic, neutral amino acids with short unbranched side chains and the synthetic amino acid α-(methylamino)isobutyric acid (MeAIB) [5]. This non-metabolised amino acid analogue has a specific affinity for system A [6] and has been used extensively to study this transport system in the placenta [4,7–9].

Molecular characterisation has revealed there are three highly homologous protein subtypes of system A; SNAT1, SNAT2, and SNAT4 [10,11] encoded by *SLC38A1*, *2* and *4*, respectively. Characterisation of these subtypes has revealed that SNAT1 and 2 are kinetically very similar [12–14], whereas SNAT4 has a relatively lower affinity for neutral amino acids and also interacts with cationic amino acids in a Na^+^-independent manner such that it resembles system y^+^L [15,16]. Each of the subtypes are expressed in human placenta [17,18], but their relative contribution to system A-mediated amino acid transport across gestation of normal pregnancy, or in FGR, is poorly understood.

The gestational changes in human placental system A activity, and the reduction in FGR, cannot be explained simply by altered expression of the three transporter subtypes [17–19], suggesting an important locus for regulation of system A is at the level of transporter activity. We have previously shown that the contribution of SNAT4 to placental system A activity is relatively high during first trimester and decreases towards term [18]. This leads us to propose that at later stages of pregnancy, SNAT1 and/or SNAT2 are responsible for the increase in placental system A activity that occurs across gestation.

The aims of our study were to identify the predominant subtype of system A in term human placenta by: (1) measuring mRNA expression of the isoforms and the determining Michaelis–Menten constants for MeAIB uptake in cytotrophoblast cells isolated from term placenta and maintained in primary culture, (2) determining the contribution of SNAT1 to total system A activity in cytotrophoblast cells using siRNA technology.

## 2 Materials and methods

### 2.1 Materials

All chemicals were purchased from Sigma–Aldrich Co. Ltd. (Poole, UK) or VWR International (Lutterworth, UK) unless otherwise stated.

### 2.2 Primary cytotrophoblast cell isolation and culture

Term placentae (38–40 weeks gestation) were collected with written informed consent and in accordance with Local Ethics Committee approval following Caesarean section or vaginal delivery from uncomplicated singleton pregnancies. Cytotrophoblast cells were then isolated using an adaptation of the method developed by Kliman et al. [20] as described previously [21]. Isolated cells were plated in culture medium (Dulbecco’s modified Eagle’s medium and Ham’s F-12 1:1, 10% heat inactivated Fetal Calf Serum, 0.6% glutamine, and antibiotics; 1% gentamicin, 0.2% penicillin, 0.2% streptomycin) onto 35-mm culture dishes (Nunc), at a density of 2–2.5 × 10^6^, and were maintained in primary culture for 66 h at 37 °C in a humidified incubator (95% air/5% CO_2_).

### 2.3 Quantitative PCR analysis of SNAT subtype mRNA expression in cytotrophoblast cells

Following 66 h in culture, cyotrophoblast cells were lysed and total RNA extracted using Absolutely RNA Microprep Kit (Stratagene, USA). RNA was quantified using Quant-iT Ribogreen kit (Molecular Probes) and 100 ng of total RNA from each sample reverse transcribed using AffinityScript cDNA synthesis kit with random primers (Stratagene, USA). mRNA for SNAT1 (*SLC38A1*), SNAT2 (*SLC38A2*), SNAT4 (*SLC38A4*), and β-actin were quantified in a 1:10 dilution of the cDNA samples by QPCR using Stratagene’s MX3000P real time PCR machine and Brilliant SYBR Green I QPCR mastermix (Stratagene, USA), with 5-carboxy-x-rhodamine as a passive reference dye. Primers (MWG-Biotech) for *SLC38A1*, *SLC38A2* and *SLC38A4* were used at a final concentration of 300 nM, and for β-actin at 200 nM as previously described [17,22]. mRNA were quantified against standard curves generated from either human liver RNA (Ambion Inc., Cambridgeshire) for *SLC38A4*, or human reference total RNA (Stratagene, La Jolla, USA) for other genes. Levels of SNAT subtype mRNA expression in cytotrophoblast cell samples were compared statistically using a non-parametric paired test, with a *p* value of <0.05 considered significant.

### 2.4 Determining the K_m_ and V_max_ of MeAIB uptake by primary cytotrophoblast cells

Following 66 h in culture, primary cytotrophoblast cells were washed free of cell culture medium using Tyrode’s buffer (135 mM NaCl, 5 mM KCl, 1.8 mM CaCl_2_, 1 mM MgCl_2_, 10 mM Hepes, 5.6 mM glucose, pH 7.4) and the uptake of radiolabelled ^14^C-MeAIB (10 nM in Tyrode’s buffer), in the presence of varying concentrations of unlabelled MeAIB (10–25 mM in Tyrode’s buffer), was measured in duplicate at 37 °C. Uptake was terminated after 20 min, a time previously confirmed to be at initial rate (data not shown), by washing cells in 25 ml ice cold Tyrode’s buffer over 1 min. Cells were then lysed in 1 ml 0.3 M NaOH and the lysate counted for β radiation. Cell lysate protein content (mg) was determined using the method of Bradford [23] using a commercial kit (Bio-Rad Laboratories Ltd.: Hemel Hampstead, UK). Uptake of radiolabelled MeAIB is expressed as pmol per mg protein over 20 min. Kinetic modelling of MeAIB competitive inhibition curves was achieved using the SIMFIT computer program (SIMFIT version 6.0.18; W.G. Bardsley, University of Manchester; http://www.simfit.man.ac.uk) which supports curve fitting of data with discrimination of kinetically distinct transporters by determining the best-fit to the Michaelis–Menten equation. The program assumes the kinetic transformation process is the same whether the substrate is labelled or not, so if the radiolabelled substrate is fixed ([hot]), the initial rate of uptake (*y*) will be proportional to the concentration of unlabelled substrate ([cold]) added, allowing isotope displacement kinetics to be modelled to the following equation:

(1) $$y=\frac{d[\text{hot}]}{\mathrm{dt}}=\frac{V_{\text{max1}}}{K_{\text{m1}}+[\text{cold}]}+\frac{V_{\text{max2}}}{K_{\text{m2}}+[\text{cold}]}$$

where [hot] = concentration of radiolabelled substrate, [cold] = concentration of unlabelled substrate, *K*_m_ = Michaelis constant and *V*_max_ = maximal velocity. The best fit to the data was determined by the *F* test, comparing the closeness of fit (weighted sum of squares of the variance) against the number of parameters in the model.

### 2.5 Transfection of primary cytotrophoblast cells with siRNA

Initial experiments using fluorescently-labelled non-targeting siRNA sequences to optimise transfection conditions, and Actinomycin D to establish the half-life of *SLC38A1* mRNA (approximately 6 h, *n* = 3), led to a protocol in which after 18 h in culture, cytotrophoblast cells were transfected with 50 or 100 nM SNAT1-specific siRNA (Qiagen) using DharmaFECT2 transfection reagent (Dharmacon) as described previously [22]. Initially, four different SNAT1-specific siRNAs were tested and here we present data using the construct which most efficiently silenced *SLC38A1* (target sequence: 5′-CAGAGCTAAATTCAACAATAA-3′). Cytotrophoblast cells transfected with non-targeting siRNA (Invitrogen) and cells exposed to DharmaFECT2 only (i.e. mock transfected) were included as controls.

### 2.6 Confirmation of target-specific knockdown

Forty-eight hours post-transfection, cells were lysed and total RNA was extracted, quantified and reverse transcribed as described above. *SLC38A1, SLC38A2*, *SLC38A4* and *β-actin* mRNA expression was analysed by quantitative PCR to confirm target-specific knockdown. Data were analysed by Wilcoxon-signed rank test following normalisation of mRNA expression to the mock-transfected control sample for the corresponding experiment.

### 2.7 System A activity measurements following SNAT1 knockdown

Forty-eight hours post-transfection, Na^+^-dependent uptake of ^14^C-MeAIB (10 nM) by control and transfected cytotrophoblast cells was measured over 20 min. Uptake of ^14^C-MeAIB was carried out at 37 °C in either control or Na^+^-free Tyrode’s buffer (135 mM choline chloride replaced NaCl, pH 7.4) using the same procedure as described above. The Na^+^-dependent component of ^14^C-MeAIB uptake, representing system A-specific uptake, was calculated by subtracting ^14^C-MeAIB uptake in the absence of Na^+^ from uptake in the presence of Na^+^. Data were analysed by Wilcoxon-signed rank test following normalisation to Na^+^-dependent ^14^C-MeAIB uptake by the corresponding control sample (i.e. untransfected) for each experiment.

## 3 Results and discussion

### 3.1 Comparison of SNAT subtype mRNA expression levels in term cytotrophoblast cells

**Fig. 1:**
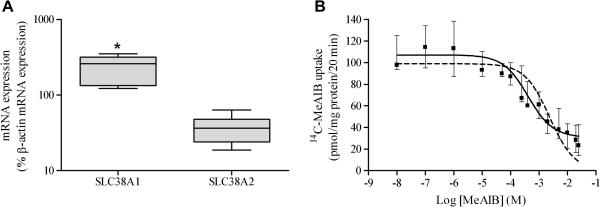
(A) Box and whisker plots to show *SLC38A1* and *A2* mRNA expression in term cytotrophoblast cells relative to β-actin mRNA expression (*n* = 7). The box denotes the interval between the 25th and 75th percentile, the whiskers represent the range, and the line inside the box shows the median; ∗*p* < 0.01, Wilcoxon-signed rank test. (B) Kinetic modelling of ^14^C-MeAIB uptake in cytotrophoblast cells, using non-linear regression analysis, with one-site (dashed line) and two-site (solid line) binding components. A two-site model was preferred (*F* test, *p* < 0.05). Data are shown as median and interquartile range (*n* = 5).

Standard curve efficiencies and *C*_t_ values were comparable for *SLC38A1* and *SLC38A2* QPCR (efficiencies: 83.4% and 83.5%, respectively). This allowed normalisation of *SLC38A1* and *SLC38A2* mRNA expression in each sample to corresponding β-actin mRNA expression, thereby correcting for any differences in reverse transcription efficiency between the samples. Fig. 1A demonstrates *SLC38A1* mRNA expression in cytotrophoblast cells was significantly higher than *SLC38A2* mRNA expression. *SLC38A4* mRNA was detected in all samples but often at a quantity below the lowest standard (0.78 ng). Therefore, *SLC38A4* mRNA data are not shown as levels were too low to accurately quantify. This observation was not unexpected as we have previously found that *SLC38A4* mRNA levels in villous tissue homogenates from some term placentas are below the levels of detection using QPCR [17].

### 3.2 Kinetics of MeAIB transport by term cytotrophoblast cells

Kinetic modelling by non-linear regression analysis of the inhibition by unlabelled MeAIB of ^14^C-MeAIB uptake into primary cytotrophoblast cells (Fig. 1B), revealed that the model of best fit was to a two saturable transporter model (*F* test, *p* < 0.05) and this trend was consistent for each of the five individual cytotrophoblast isolates (*r*^2^ ⩾ 0.83). The kinetic characteristics for each transport system were; system 1: *K*_m_ = 0.38 ± 0.12 mM, *V*_max_ = 27.8 ± 9.0 pmol/mg protein/20 min, and system 2: *K*_m_ = 45.4 ± 25.0 mM, *V*_max_ = 1190 ± 291 pmol/mg protein/20 min. The kinetic characteristics of the two systems identified indicate there is a high affinity, low capacity transport system for MeAIB in these cells (system 1) as well as a low affinity, high capacity transport system (system 2). As all three system A subtypes are expressed by these cells, it is possible that two have a very similar affinity for MeAIB. Experiments using human retinal pigment epithelial (HRPE) cells manipulated to express the individual system A subtypes revealed *K*_m_ values for SNAT1, SNAT2 and SNAT4 of 0.89, 0.39, 6.7 mM, respectively [12,13,15]. Bearing in mind the potential differences in intracellular regulatory factors and/or plasma membrane composition between cell types which could influence transporter kinetics, the *K*_m_ value of system 1 determined here is reasonably similar that reported for SNAT1 and SNAT2 in HRPE cells and therefore, could represent transport by both these system A subtypes. We propose that system 2 represents SNAT4 despite the *K*_m_ value being almost 7-fold higher than that reported for SNAT4 in HRPE cells. Again, this could reflect differences in lipid environment of the transporter between the cell types. Indeed, the *K*_m_ for taurine transport by system β varies quite considerably between different cell types from the same species (in some cases over 10-fold) and between particular cell types isolated from different species [24].

### 3.3 SNAT1-specific knockdown in primary cytotrophoblast cells

**Fig. 2:**
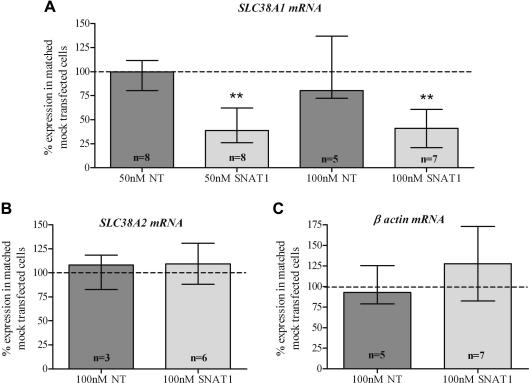
(A) *SLC38A1*, (B) *SLC38A2*, and (C) *β-actin* mRNA expression in cells transfected with non-targeting (NT) or *SLC38A1*-specfic (SNAT1) siRNA relative to corresponding mock transfected cytotrophoblast cells (dashed line at 100%). Median and interquartile range; ∗∗*p* < 0.01 vs 100%, Wilcoxon-signed rank test.

Following transfection of cytotrophoblast cells with 50 and 100 nM SNAT1-specific siRNA, *SLC38A1* mRNA expression was significantly reduced compared to mock-transfected cells (Fig. 2A). siRNA (50 and 100 nM) were equally effective in eliciting knockdown of *SLC38A1* mRNA, indicating the lower concentration was sufficient to achieve maximal knockdown. Transfection of cells with non-targeting siRNA did not affect *SLC38A1* mRNA eliminating the possibility that simply delivering siRNA into the cell causes reduced *SLC38A1* mRNA expression (Fig. 2A). *SLC38A2* and β-actin mRNA expression was unaffected following transfection of cells with 100 nM SNAT1-specific siRNA (Fig. 2B and C) confirming specificity of the siRNA for its target.

### 3.4 Contribution of SNAT1 to system A activity in term cytotrophoblast cells

**Fig. 3:**
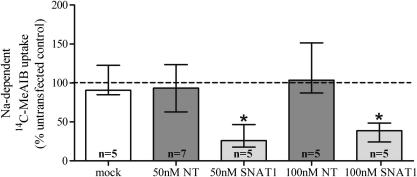
System A activity, measured as Na^+^-dependent ^14^C-MeAIB uptake, by mock transfected cytotrophoblast cells and cells transfected with non-targeting (NT) or *SLC38A1*-specific (SNAT1) siRNA. System A activity is expressed relative to the corresponding untransfected control (dashed line at 100%). Median and interquartile range; ∗*p* < 0.05 vs 100%, Wilcoxon-signed rank test.

Knockdown of *SLC38A1* mRNA in cytotrophoblast cells was accompanied by a significant reduction in system A activity, measured as Na^+^-dependent uptake of ^14^C-MeAIB (Fig. 3). In common with the mRNA data, transfection with 50 nM SNAT1-specific siRNA produced a similar effect to transfection with 100 nM siRNA, further confirming maximal knockdown was achieved using the lower concentration of siRNA. As shown in Table 1, 50 nM SNAT1-specific siRNA did not affect the Na^+^-independent component of ^14^C-MeAIB uptake (i.e. that not mediated by system A). System A activity in cytotrophoblast cells was unaffected following treatment with DharmaFECT2 transfection reagent or transfection with non-targeting siRNA (Fig. 3).

Data presented in Fig. 2A demonstrate that complete knockdown of the SNAT1 transporter mRNA was not achievable using 50 nor 100 nM SNAT1-specific siRNA as some residual expression remained. Therefore, it is possible that a small proportion of the system A activity observed in cells transfected with SNAT1-specific siRNA (shown in Fig. 3) represents SNAT1-mediated MeAIB transport, with the remaining majority representing SNAT2-mediated MeAIB transport. The contribution of SNAT4 is likely to be minimal based on the relatively low levels of *SLC38A4* expression in cytotrophoblast cells and the lower affinity of SNAT4 for MeAIB compared to SNAT2 [12,15].

In central neurons, the selectivity of SNAT1 for MeAIB is less than one-tenth of that for its preferred substrates, alanine or glutamine [25], and therefore, MeAIB is perhaps not an ideal substrate for use in experiments designed to investigate SNAT1 activity. However, as MeAIB is a specific substrate for system A and, in the present study, MeAIB was the only substrate available, it would have been readily transported by any system A subtype. Considering the affinity of SNAT2 in HRPE for MeAIB is similar to that for its preferred substrate, alanine [12], it has perhaps been reasonable to assume that system A activity measurements previously made in placenta using MeAIB would mainly represent SNAT2-mediated transport. However, our data show that under the conditions of these experiments, SNAT1 made the major contribution to system A activity in cytotrophoblast cells isolated from term human placenta.

Reduced expression and/or activity of SNAT1 in the placenta could, therefore, underlie the compromised system A activity in placentas from pregnancies complicated with FGR [3]. The regulatory factors responsible for altered placental system A activity in cases of FGR are currently under investigation and there is evidence to suggest these include oxygen tension, substrate levels, as well as some cytokines, hormones and growth factors [1,26]. Each of these factors are altered in pregnancy complications associated with placental pathology and could, therefore, influence system A activity through differential effects on the SNAT subtypes.

## 4 Conclusions

Our data contribute to the limited understanding of the relative importance of the three system A subtypes for amino acid transport in human placenta by demonstrating that knockdown of the SNAT1 subtype in primary cytotrophoblast cells significantly reduces MeAIB uptake, thereby suggesting that SNAT1 is a key contributor to term placental system A activity. However, all three subtypes are present in placenta, which implies there is an important biological role for each. Accumulating evidence suggests there is differential regulation of the three SNAT subtypes [27–29]. Therefore, future studies investigating system A in the placenta should continue to focus on the differential regulation of the three SNAT subtypes in parallel to transporter activity measurements. This will help to further elucidate the relative roles of each subtype for system A-mediated amino acid transport and facilitate our understanding of the processes involved in those alterations associated with pathological pregnancy conditions.

## Figures and Tables

**Table 1:** Components of ^14^C-MeAIB uptake by transfected cytotrophoblast cells.

	Median (range) MeAIB uptake (% of untransfected control)		
	Total	Na^+^-independent	Na^+^-dependent
50 nM NT	91.5 (51.1–127.8)	81.34 (54.1–180.3)	93.5 (49.9–126.7)
50 nM SNAT1	47.8 (25.2–70.1)⁎	113.7 (81.5–222.8)	25.9 (13.5–62.9)⁎

⁎ *p* < 0.05 vs 100%, Wilcoxon-signed rank test.
